# Metabolic shift toward oxidative phosphorylation in docetaxel resistant prostate cancer cells

**DOI:** 10.18632/oncotarget.11301

**Published:** 2016-08-16

**Authors:** Luigi Ippolito, Alberto Marini, Lorenzo Cavallini, Andrea Morandi, Laura Pietrovito, Gianfranco Pintus, Elisa Giannoni, Thomas Schrader, Martin Puhr, Paola Chiarugi, Maria Letizia Taddei

**Affiliations:** ^1^ Department of Experimental and Clinical Biomedical Sciences, University of Florence, Florence, Italy; ^2^ Department of Chemistry, University Duisburg-Essen, Essen, Germany; ^3^ Department of Urology, Medical University of Innsbruck, Innsbruck, Austria; ^4^ Tuscany Tumor Institute and “Center for Research, Transfer and High Education DenoTHE”, Florence, Italy; ^5^ Department of Biomedical Sciences, Laboratory of Cell Signaling and Redox Biology, University of Sassari, Sassari, Italy; ^6^ Department of Biomedical Sciences, College of Health Sciences, Qatar University, Doha, Qatar; ^7^ Department of Experimental and Clinical Medicine, University of Florence, Florence, Italy

**Keywords:** prostate cancer, chemoresistance, docetaxel, oxidative phosphorylation, epithelial mesenchymal transition

## Abstract

Drug resistance of cancer cells is recognized as the primary cause of failure of chemotherapeutic treatment in most human cancers. Growing evidences support the idea that deregulated cellular metabolism is linked to such resistance. Indeed, both components of the glycolytic and mitochondrial pathways are involved in altered metabolism linked to chemoresistance of several cancers. Here we investigated the drug-induced metabolic adaptations able to confer advantages to docetaxel resistant prostate cancer (PCa) cells. We found that docetaxel-resistant PC3 cells (PC3-DR) acquire a pro-invasive behavior undergoing epithelial-to-mesenchymal-transition (EMT) and a decrease of both intracellular ROS and cell growth. Metabolic analyses revealed that PC3-DR cells have a more efficient respiratory phenotype than sensitive cells, involving utilization of glucose, glutamine and lactate by the mitochondrial oxidative phosphorylation (OXPHOS). Consequently, targeting mitochondrial complex I by metformin administration, impairs proliferation and invasiveness of PC3-DR cells without effects on parental cells. Furthermore, stromal fibroblasts, which cause a “reverse Warburg” phenotype in PCa cells, reduce docetaxel toxicity in both sensitive and resistant PCa cells. However, re-expression of miR-205, a microRNA strongly down-regulated in EMT and associated to docetaxel resistance, is able to shift OXPHOS to a Warburg metabolism, thereby resulting in an elevated docetaxel toxicity in PCa cells. Taken together, these findings suggest that resistance to docetaxel induces a shift from Warburg to OXPHOS, mandatory for conferring a survival advantage to resistant cells, suggesting that impairing such metabolic reprogramming could be a successful therapeutic approach.

## INTRODUCTION

Docetaxel (Taxotere^®^) is a standard chemotherapy for patients with castration resistant metastatic prostate cancer [1, 2]. However, docetaxel-based chemotherapy often encounters several undesirable adverse effects, and many patients display *de novo* or acquired resistance. To date, several factors have been associated with docetaxel resistance, including expression of different isoforms of β-tubulin [3], activation of drug efflux pumps [4], PTEN loss [5], activation of survival pathways (i.e., PI3K/AKT and mTOR) [6] and recently also NOTCH2/Hedgehog signaling pathways [7]. Recent findings both *in vitro* and from tumor samples support the presence of primary resistant cells harboring EMT/stem cell–like characteristics [8] suggesting a possible association between such aggressive features and chemotherapy failure. Interestingly, acquisition of metastatic characteristics is also associated with a specific metabolic reprogramming [9] and tumor metabolism has received increased attention over the last decade. Only recently the metabolic behavior has been implicated in tumor drug resistance [10–12]. Targeting tumor metabolism has been shown to represent an alternative way to overcome drug resistance and there are several approaches that have been demonstrated to be successful in pre-clinical models [13, 14]. However, the link between tumor metabolism and drug resistance is highly complex and depends on various conditions including oxygen and/or nutrient availability [15, 16] and can be influenced by the surrounding microenvironment [17]. Indeed, in tumor microenvironment, cancer-associated fibroblasts (CAFs) have been shown to promote aggressiveness of PCa cells in terms of EMT induction [18], OXPHOS metabolic shift [19, 20] and miRNAs deregulation [21]. In this study, we report a metabolic shift of docetaxel-resistant PCa cells from a glycolytic phenotype towards OXPHOS due to EMT engagement. We also demonstrate that CAFs are able to protect tumor cells from drug toxicity. Finally, in agreement with recent results highlighting the key role of microRNA in tumor progression [22], we focused our attention on miR-205 which is down-regulated in both CAF and docetaxel induced EMT [8, 21]. We demonstrated that overexpression of miR-205, associated with a reversion of OXPHOS metabolism, is crucial to sensitize PC3-DR to the drug.

## RESULTS

### PC3-DR cells acquire pro-invasive abilities and show decreased levels of ROS and pentose phosphate pathway flux

We established the PC3-DR cell line by treating sensitive PC3 cells with increasing doses of docetaxel up to a final concentration of 10 nM. PC3-DR cells achieve EMT as shown by cell morphology, EMT markers, increased cell invasion and secretion of interleukin-6 (IL6), a marker of prostate cancer progression [23] (Supplementary Figure 1A-1E). Furthermore, PC3-DR cells show a decreased expression of several pro-apoptotic markers as well as an increased clonogenic potential as assessed by prostaspheres formation assay (Supplementary Figure 1E-1F).

**Figure 1:**
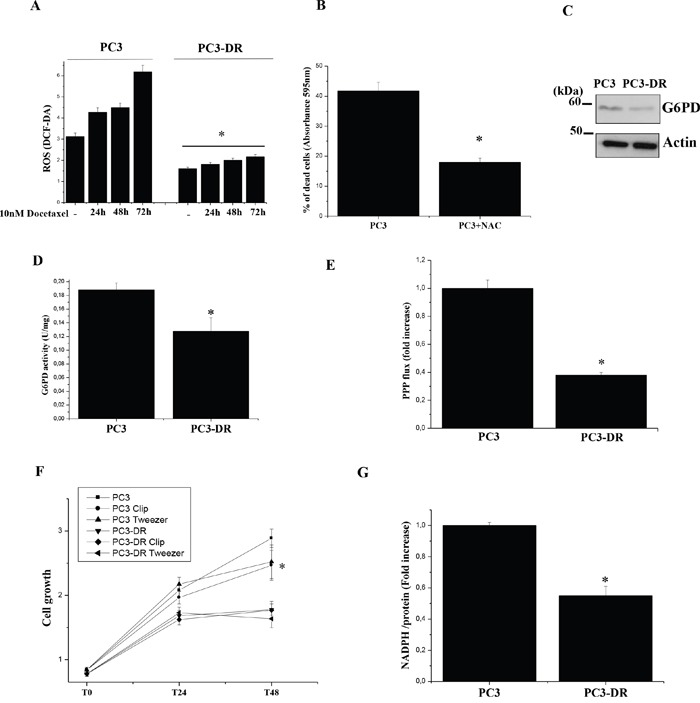
PC3-DR cells have reduced levels of intracellular ROS, PPP flux and cell growth **A.** Evaluation of intracellular ROS with H_2_DCF-DA fluorimetric analysis, normalized on protein content. The results are representative of four independent experiments. *p≤0.005 PC3-DR *vs* PC3 **B.** Evaluation of PC3 cells sensitivity to docetaxel in presence of NAC: PC3 cells were treated with 5 nM docetaxel for 24 h in the presence or not of 20 mM NAC. Cells viability was quantified by crystal violet assay. Bar graph represents the percentage of dead cells following treatments with respect to corresponding untreated cells. Results are representative of three experiments. *p<0.005 NAC *vs* untreated. **C.** Immunoblot analysis of G6PD expression levels in PC3 and PC3-DR cell lines. Actin immunoblot was used for normalization. **D.** G6PD activity assay in PC3 and PC3-DR cells. The results are representative of three experiments. *p≤0.05 PC3-DR *vs* PC3. **E.** Analysis of PPP flux was obtained by subtracting the amount of CO_2_ developed from [6-^14^C]-glucose from the CO_2_ released from [1-^14^C]-glucose. The value is normalized on the protein content and expressed as fold change relative to PC3 cells. The results are representative of three experiments. *p≤0.005 PC3-DR *vs* PC3. **F.** Cell growth of PC3 and PC3-DR cells was evaluated by crystal violet staining in complete medium in the presence or not of 10 μM Molecular Clip or 3 μM Tweezer added fresh daily. *p≤0.005 PC3-DR *vs* PC3. **G.** Quantification of NADPH intracellular levels of PC3 and PC3-DR cells reported as fold change relative to PC3 cells. The results are representative of three experiments. *p≤0.005 PC3-DR *vs* PC3.

Recently, it has been demonstrated that docetaxel treatment elicits a burst of ROS produced by NADPH oxidase [24]. Indeed, enhancement of ROS production is associated to many chemotherapeutic agents [24–27]. Therefore, the ability of cancer cells to handle oxidative stress is fundamental for the protection of cells against the cytotoxic effect of anti-cancer agents and hence for the development of chemoresistance. To gain insights on this aspect, we evaluated the ability of PC3-DR and PC3 cells to manage ROS. As demonstrated in Figure 1A PC3-DR cells have reduced ROS levels both in basal condition and following docetaxel treatment with respect to PC3 sensitive cells. In agreement, treatment of sensitive PC3 cells with the ROS scavenger N-acetylcysteine (NAC) decreases their sensitivity to docetaxel (Figure 1B). Recently, the activation of the pentose phosphate pathway (PPP) has been implicated in chemoresistance of cancer cells [28–30] through the production of NADPH required to fuel antioxidant systems. We evaluated the expression and activity of the key PPP rate limiting enzyme glucose-6-phosphate dehydrogenase (G6PD) as well as PPP flux by radioactive assay in PC3-DR and in sensitive cells (Figure 1C-1E). Surprisingly, we found a reduced expression and activity of G6PD (Figure 1C-1D) and a reduced PPP flux in the resistant cells (Figure 1E). Moreover, PC3-DR cells are insensitive to G6PD inhibitors, Molecular Clip and Tweezer [31] (Figure 1F), suggesting that docetaxel-resistance does not depend on PPP activation in the model analyzed. Furthermore, NADPH levels are decreased in PC3-DR cells (Figure 1G). These results suggest that in the PC3 model PPP is not involved in counteracting oxidative stress induced by the drug. Interestingly, in agreement with a reduction of PPP flux, which is fundamental not only for NADPH synthesis but also for ribose 5 phosphate production to sustain nucleic acid synthesis and hence proliferation, we observed a decrease in cell growth of PC3-DR with respect to PC3 (Figure 1F).

### Docetaxel resistant cells acquire a respiratory phenotype

**Figure 2:**
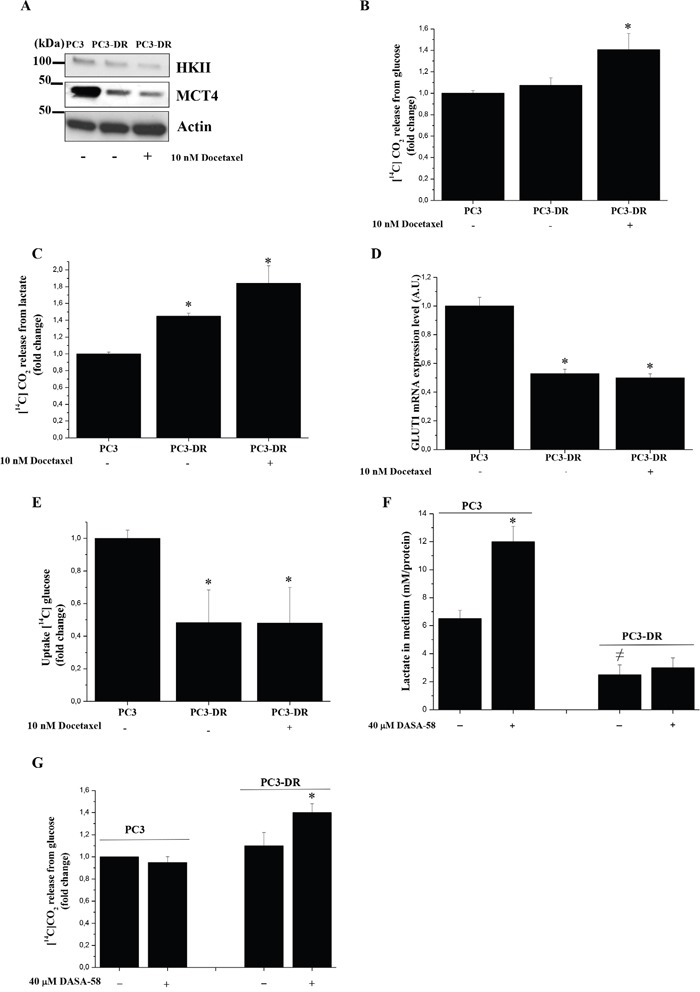
Docetaxel resistant cells increase their oxidative metabolism **A.** Immunoblot analysis of HKII and MCT4 in PC3 and PC3-DR cells treated with or without 10 nM docetaxel in serum-free medium for 48 h. Actin immunoblot to ensure equal loading. **B.** Respiration of [^14^C]-glucose of PC3 and PC3-DR cells treated as in A) was evaluated by monitoring the [^14^C]-CO_2_ release and normalized on protein content. Results are shown as fold change relative to PC3 cells. The results are representative of four experiments. *p<0.05 PC3-DR *vs* PC3. **C.** Respiration of [^14^C]-lactate of PC3 and PC3-DR cells treated as in A) was evaluated by monitoring the [^14^C]-CO_2_ release and normalized on protein content. Results are shown as fold change relative to PC3 cells. The results are representative of three experiments. *p<0.01 PC3-DR *vs* PC3. **D.** qRT-PCR analysis of GLUT1 expression in PC3 and PC3-DR cells. Results are representative of three experiments. *p≤0.005 PC3-DR *vs* PC3. **E.** Evaluation of [^14^C]-glucose uptake was performed and normalized on protein content. Results are representative of three experiments. *p≤0.05 PC3-DR *vs* PC3. **F.** Cells were treated in the presence or absence of 40 μM DASA-58 for 48 h in serum free medium. The amount of lactate released in the medium was quantified and plotted after protein normalization. Results are representative of three experiments. *p<0.01 *vs* PC3 without DASA-58; ≠p<0.01 *vs* PC3 without DASA-58. **G.** Respiration of [^14^C]-glucose of PC3 and PC3-DR cells treated as in F) was evaluated as [^14^C]-CO_2_ release and normalized on protein content. Results are representative of three experiments. * p<0.005 *vs* PC3-DR without DASA-58.

To establish a potential correlation between docetaxel resistance and metabolic reprogramming, we analyzed proteins/enzymes involved in aerobic glycolysis and OXPHOS. To understand the metabolic adaptations of resistant cells following treatment with the drug and in order to reproduce circumstances occurring *in vivo* during treatment with the anti-cancer agent, we performed the experiments even in the presence of an acute treatment of docetaxel. As shown in Figure 2A PC3 cells have higher levels of Hexokinase II (HKII) and monocarboxylate transporter 4 (MCT4), that have been shown to be associated with Warburg metabolism, when compared to PC3-DR cells, independently of docetaxel treatment, suggesting that docetaxel resistance leads to an adaptation towards mitochondrial metabolism. Indeed, analysis of radiolabeled carbon sources fluxes shows that docetaxel resistant cells exhibit an increased glucose and lactate oxidation to CO_2_ through mitochondrial respiration (Figure 2B-2C) accompanied by a decrease in GLUT1 expression and glucose uptake (Figure 2D-2E). To sustain the key role of OXPHOS in supporting chemoresistance, we demonstrated that in hypoxic conditions, where both HKII and MCT4 expression increases, thus restoring Warburg metabolism, PC3-DR cells become more sensitive to docetaxel (Supplementary Figure 2). In agreement, another prostate cell line, namely the DU-145, exhibits a similar respiratory phenotype after acquisition of docetaxel resistance and mesenchymal phenotype as shown by increased levels of glucose and lactate oxidation and a decrease in glucose uptake; in keeping DU-145-DR cells show an increase in c-Myc expression (a key regulator of mitochondrial respiration) and a decrease in HKII levels (Supplementary Figure 3A-3C). Taken together, these data support the existence of a shift towards the oxidative mitochondrial metabolism to sustain the acquisition of the docetaxel resistance phenotype.

**Figure 3:**
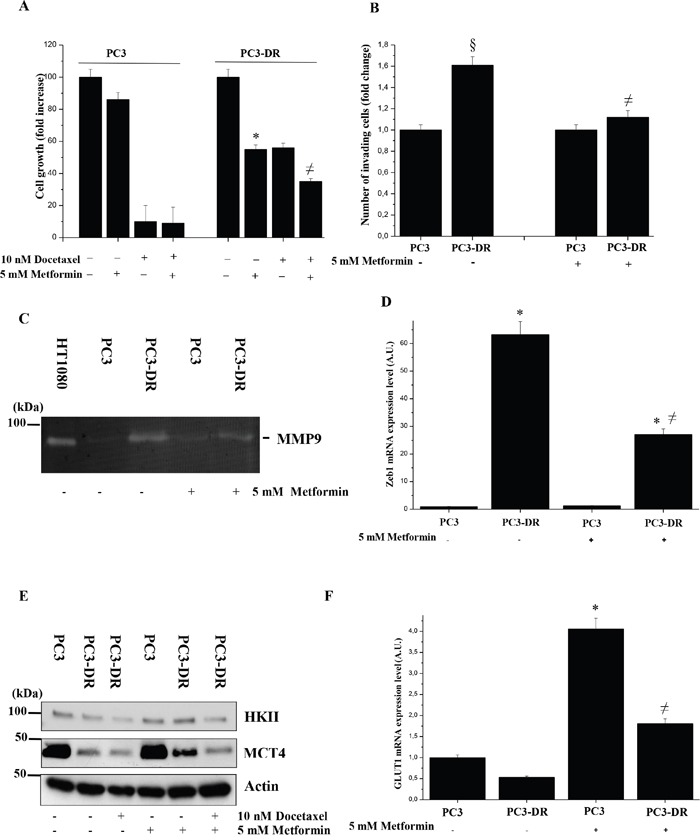
Metformin impairs growth and invasion of PC3-DR cells **A.** Analysis of PC3 and PC3-DR cells growth in 10 % FBS medium for 48 h in presence of 10 nM docetaxel and/or 5 mM metformin by crystal violet assay. Absorbance at T0 of untreated PC3 and untreated PC3-DR cells was used as control. The results are representative of three experiments. *p<0.005 Metformin treated *vs* untreated. **B.** Boyden invasion assay of PC3 and PC3-DR cells treated or not with 5 mM metformin. Bar graph represents the mean of invaded cells in six-fields/sample. Number of invaded cells was expressed as fold change relative to PC3 cells without metformin. §p<0.05 *vs* PC3 without metformin; ≠p<0.05 *vs* PC3-DR without metformin. **C.** MMPs activity in PC3 and PC3-DR cells, treated or not with 5 mM metformin, was evaluated by gelatine zymography. Clear bands represent areas of gelatinase activity. The results shown are representative of four independent experiments with comparable results. Medium from HT1080 cell was used as positive control. **D.** qRT-PCR analysis of Zeb1 expression was analyzed. Results are representative of three experiments. ≠p<0.005 metformin treated *vs* untreated; *p<0.005 PC3-DR *vs* PC3. **E.** Immunoblot analysis of HKII and MCT4 of total cell lysates from PC3 and PC3-DR cells treated or not with 5 mM metformin and/or 10 nM docetaxel for 48 h in serum-free medium. Actin immunoblot was used for normalization. **F.** qRT-PCR analysis of GLUT1 expression levels was analyzed. Results are representative of three experiments. *p<0.005 metformin treated *vs* untreated PC3; ≠p<0.005 metformin treated *vs* PC3-DR.

To deep analyze the fate of pyruvate produced in PC3-DR and PC3 cells, we administrated DASA-58, a molecule that induces pyruvate kinase M2 (PKM2) tetramerization (hence activation) and thus pyruvate production [32]. Upon DASA-58 administration we demonstrated that resistant and sensitive cells show differential behavior: PC3 cells, through the conversion of pyruvate into lactate, show a marked increase of lactate levels in the medium, while PC3-DR cells do not change their lactate production (Figure 2F), but increase glucose-fueled oxidation through OXPHOS (Figure 2G). To better clarify the engagement of mitochondrial respiration in docetaxel resistant cells, we used metformin, an inhibitor of the mitochondrial complex I activity, to test its effect on both cell growth and invasion of these two cell types. As shown in Figure 3A-3D in docetaxel resistant cells metformin selectively inhibits growth, invasiveness, MMPs secretion and Zeb1 expression. These effects are correlated with the disruption of the mitochondrial respiratory function, suggesting that PC3-DR cells are likely dependent on it. Indeed, metformin causes a change in the metabolic behaviour of cancer cells as shown by the upregulation of HKII, MCT4 and GLUT1 expression thus shifting the metabolism towards a clear glycolytic one and consequently impairing the features of mitochondrial dependent cells (Figure 3E-3F). To further support the involvement of mitochondrial respiration in docetaxel resistant cells we used other OXPHOS inhibitors (rotenone, antimycin and oligomycin) to assess the sensitivity to docetaxel. As shown in Supplementary Figure 4, OXPHOS inhibitors affect more efficiently PC3-DR survival, confirming a strict connection between resistance and mitochondrial respiration.

**Figure 4:**
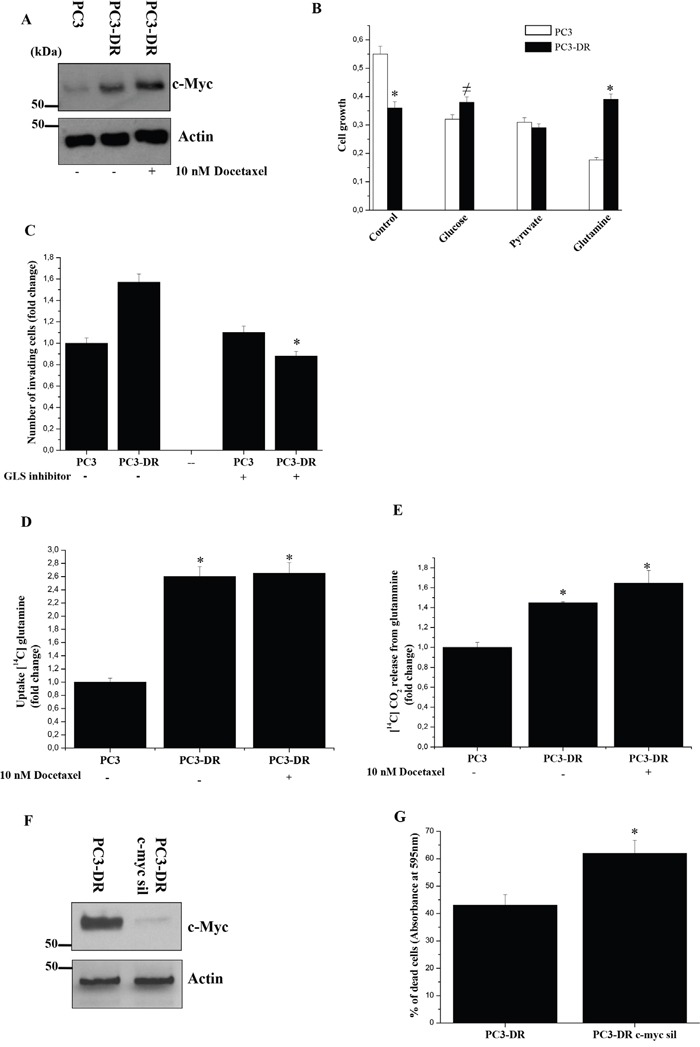
PC3-DR cells acquire glutamine addiction **A.** Immunoblot analysis of c-Myc in PC3 and PC3-DR cells treated with or without 10 nM docetaxel for 48 h in serum-free medium. Actin immunoblot was used for normalization. **B.** Evaluation of PC3 and PC3-DR cells growth in medium containing either glucose (25 mM), pyruvate (1 mM) or glutamine (2 mM) was analyzed by crystal violet assay for 48 h. Results are representative of three experiments. *p<0.005 PC3-DR *vs* PC3; ≠p<0.05 PC3-DR *vs* PC3. **C.** Boyden invasion assay of PC3 and PC3-DR cells treated or not with 10 μM glutaminase inhibitor (compound 968). Bar graph represents the mean of invaded cells in six field for sample. Number of invaded cells was expressed as fold change respect to PC3 cells without metformin. Results are representative of three independent experiments. *p<0.005 treated *vs* untreated. **D.** Evaluation of [^14^C]-glutamine uptake of PC3 and PC3-DR cells treated or not with 10 nM docetaxel for 48 h and normalized on protein content. Results are representative of three experiments. *p<0.005 *vs* PC3. **E.** Respiration of [^14^C]-glutamine in PC3 and PC3-DR cells treated as in D) was evaluated as [^14^C]-CO_2_ release and normalized on total protein content. Results are shown as fold change relative to PC3 cells. Results are representative of four experiments. *p<0.005 *vs* PC3. **F.** Immunoblot analysis of c-Myc in PC3-DR cells silenced with control siRNA or c-Myc siRNA after 48 h from transfection. Actin immunoblot was used for normalization. **G.** PC3-DR cells were transfected with control siRNA or c-Myc siRNA. After 24 h from transfection cells were treated or not with 20 nM docetaxel for 48 h. Cells viability was then quantified by crystal violet assay. Bar graph represents the percentage of dead cells following docetaxel treatment with respect to untreated cells. Results are representative of three experiments. *p<0.005 c-myc sil PC3-DR *vs* control PC3-DR.

It has been shown that cancer cells are also dependent on glutamine to maintain the TCA cycle [33]. Moreover, it has been demonstrated that c-Myc is implicated in glutamine metabolism and contributes to metabolic reprogramming essential for cancer cells to adapt to the tumour microenvironment, being involved also in ribosomal and mitochondrial biogenesis, glucose metabolism as well as lipid synthesis [34]. Interestingly, we found a strong up-regulation of c-Myc in PC3-DR cells (Figure 4A). To test the dependence of PC3-DR cells on glutamine, we investigated cell growth in the presence of a single carbon source in the medium. As shown in Figure 4B, PC3-DR cells exhibit higher glutamine-fuelled growth, compared to parental cells. Furthermore, inhibition of glutaminase, the key enzyme for mitochondrial glutamine utilization, strongly affects the invasive abilities of resistant cells (Figure 4C). In line with these data, PC3-DR cells exhibit an increased glutamine uptake and oxidation through OXPHOS (Figure 4D-4E). These data are consistent with both an increased mitochondrial metabolism and the acquisition of glutamine dependence in docetaxel resistant cells. Accordingly, c-Myc silencing is able to sensitize PC3-DR cells to docetaxel as shown in Figure 4F-4G, suggesting a strong dependence of PC3-DR cells on c-Myc axis.

**Figure 5:**
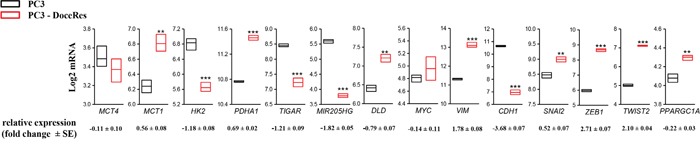
Gene expression analysis of PC3 and docetaxel-resistant derivatives GDS3973 data [35] were retrieved as described in Material and Methods and transcripts of relevant genes involved in central carbon metabolism and EMT process were monitored. Box plots represent min, max and median value. Relative mRNA changes (fold increase PC3-DR *vs* PC3) are reported. **p<0.01 *vs* PC3, ***p<0.001 *vs* PC3.

Metabolic reprogramming towards OXPHOS and the acquisition of mesenchymal traits of the PC3-DR cells was further supported by data mining of publicly available gene expression profile of PCa cells that display resistance to docetaxel [35]. In such a model, a class of genes associated with OXPHOS metabolism are upregulated in the docetaxel resistant cell line: pyruvate dehydrogenase (E1) α subunit gene (PDHA1), monocarboxylate transporter 1 (MCT1), Dihydrolipoamide dehydrogenase (DLD), MYC, Peroxisome proliferator-activated receptor gamma coactivator 1-alpha (PPARGC1A); whereas, genes found to be associated with aerobic glycolysis are down-regulated: MCT4, HK2, TP53 Induced Glycolysis Regulatory Phosphatase (TIGAR), when compared to wild type PC3 cells. Additionally, this *in silico* analysis confirmed the acquisition of mesenchymal traits of PC3 cells resistant to docetaxel. Indeed, vimentin, Snai2, Zeb1 and Twist2 were upregulated and Cdh1 down-regulated in resistant cells when compared to wild type PC3. Noteworthy, miR-205, an established regulator of EMT engagement in PC3 cells [8, 21], was found accordingly down-regulated (Figure 5).

### Cancer associated fibroblasts induce docetaxel resistance in PCa cells

**Figure 6:**
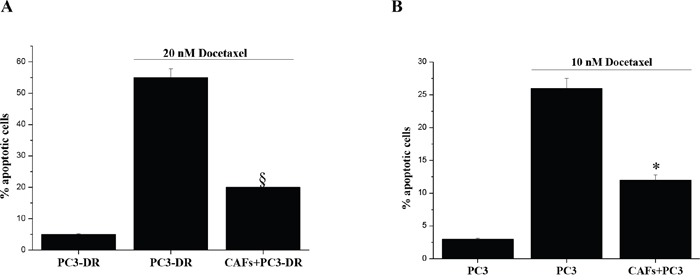
CAFs reduce sensitivity to docetaxel-induced apoptosis **A.** PC3-DR cells were cultured alone or co-cultured with CAFs (ratio CAFs:PC3 2:1) for 48 h in complete medium prior to 20 nM docetaxel treatment for additional 72 h. PCa cells were then isolated using immunomagnetic beads targeting fibroblasts and stained with Annexin V and Propidium Iodide and apoptosis was evaluated by flow cytometer. §p<0.01*vs* PC3-DR cells alone treated with docetaxel. **B.** Analysis of docetaxel-induced apoptosis with Annexin V/Propidium Iodide cytofluorimetric staining. PC3 cells were cultured alone or co-cultured with CAFs (proportion CAFs:PC3 2:1) for 48 h prior to 10 nM docetaxel treatment for further 72 h. PC3 cells were then negatively isolated using immunomagnetic beads targeting fibroblasts and stained with Annexin V and Propidium Iodide and apoptosis was evaluated by flow cytometry. *p<0.01 *vs* PC3 cells alone treated with docetaxel.

In the context of prostate cancer progression we demonstrated that OXPHOS induction is a common feature of both CAF induced metabolic reprogramming and miR-205 down-regulation [19–21] as well as drug resistance. Thus, we investigated the role of CAF and miR-205 modulation on chemosensitivity of PC3 cells. To this end we first tested whether stromal fibroblasts could affect PC3 cells chemosensitivity to docetaxel. Indeed, stromal cells are effective in supporting the survival of PC3-DR cells (10 nM resistant) when treated with an increased concentration of the drug (20 nM) (Figure 6A). Moreover, we observed that CAFs promote also the onset of PC3 cells resistance to docetaxel treatment as shown by co-culturing sensitive PC3 cells with CAFs in the presence of docetaxel (Figure 6B). Therefore, we suggest that the stromal compartment is able to counteract docetaxel toxicity through the establishment of an oxidative metabolic phenotype.

**Figure 7:**
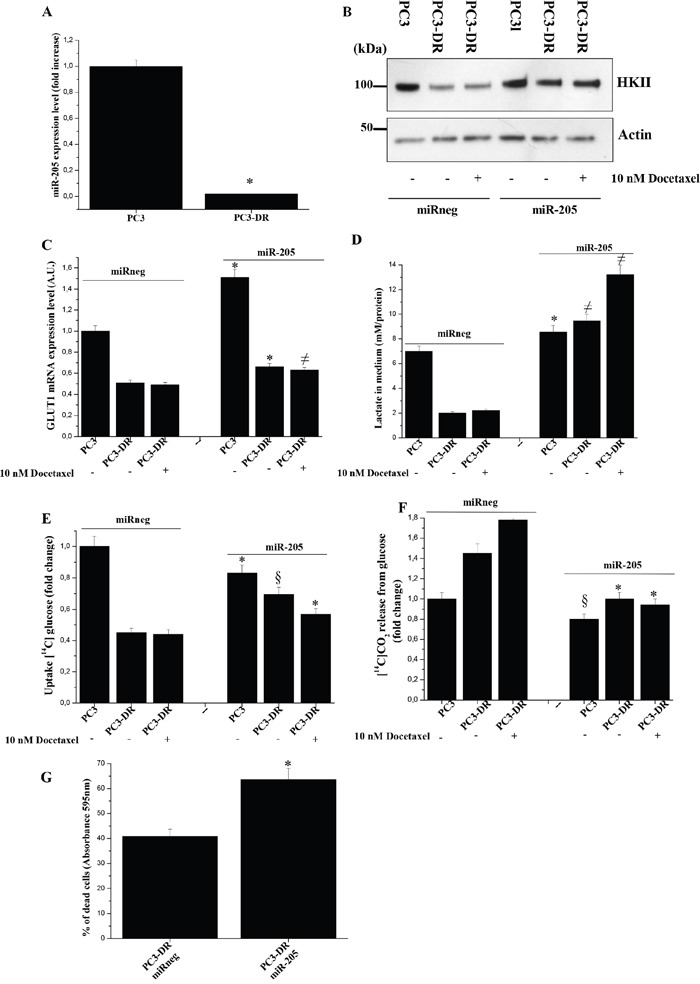
Re-expression of miR-205 in PC3-DR cells induces a metabolic shift and increase docetaxel sensitivity **A.** qRT-PCR analysis of *miR-205* expression in PC3 and PC3-DR cells. Results are representative of three experiments. *p<0.005 PC3-DR *vs* PC3. **B.** Immunoblot analysis of HKII of PC3 and PC3-DR transfected with *miR-205* or miR-neg for 48 h and then treated with or without 10 nM docetaxel for 48 h in serum-free medium. Actin immunoblot was used for normalization. **C.** qRT-PCR analysis of GLUT1 expression was evaluated in cells as in B). Results are representative of three experiments. *p<0.005 miR-205 transfected *vs* miR-neg transfected; ≠p<0.01 miR-205 transfected *vs* miR-neg transfected. **D.** Cell were treated as in B) and lactate in media was evaluated. *p<0.01 miR-205 transfected *vs* miR-neg transfected; ≠p<0.005 miR-205 transfected *vs* miR-neg transfected. **E.** Evaluation of [^14^C]-glucose uptake was performed in PC3 and PC3-DR cells treated as in B) and normalized on protein content. Results are representative of three experiments. *p<0.01 miR-205 transfected *vs* miR-neg transfected; §p<0.001 miR-205 transfected *vs* miR-neg transfected. **F.** Respiration of [^14^C]-glucose of PC3 and PC3-DR cells treated as in B) was evaluated as [^14^C]-CO_2_ release and normalized on protein content. Results are representative of three experiments. *p<0.005 miR-205 transfected *vs* miR-neg transfected; §p<0.05 miR-205 transfected *vs* miR-neg transfected. **G.** PC3-DR cells were transfected with *miR-205* or miR-neg for 24 h and then treated or not with 20 nM docetaxel for 48 h. Cells were then quantified by crystal violet assay. Bar graph represents the percentage of dead cells following docetaxel treatment with respect to untreated cells. Results are representative of four experiments. *p<0.005 miR-205 transfected PC3-DR *vs* miR-neg transfected PC3-DR.

In our previous reports we demonstrated that miR-205 is one of the most down-regulated miRNA in PCa cells undergoing EMT and OXPHOS induction upon CAF contact [20, 21]. In our model we observed that OXPHOS engagement of PC3-DR cells is correlated to a down-regulation of miR-205 expression with respect to PC3 cells, too (Figure 7A). Thus, we evaluated whether miR-205 re-expression could change the metabolic behavior of PCa cells. Indeed, ectopic miR-205 expression results in increased HKII expression and GLUT1 mRNA levels, as well as an increase in lactate production (Supplementary Figure 5 and Figure 7B-7D), suggesting an elevated glycolytic flux. Furthermore, an increase of glucose uptake and a decrease of mitochondrial glucose oxidation are observed following miR-205 overexpression in PC3-DR cells (Figure 7E-7F).

Finally, we evaluated whether this shift towards the glycolysis could be associated with increased chemosensitivity of PC3-DR cells. As expected, ectopic overexpression of miR-205 in PC3-DR cells sensitizes resistant cells to docetaxel treatment (Figure 7G). Similar results were obtained also in DU145-DR cells (Supplementary Figure 6).

## DISCUSSION

It is well known that cancer cells are able to rewire their metabolism and energy production networks to support and enable rapid proliferation, for survival in severe conditions and for increased invasion, metastasis and chemoresistance [12, 36–38]. Our results highlight that docetaxel resistant prostate cancer cells plastically shift their metabolism from aerobic glycolysis to OXPHOS and that metabolic adaptation is related to the commitment of EMT, increased invasion and achievement of stem-like traits. Furthermore, the acquisition of docetaxel resistance is associated with down-regulation of miR-205, whose reduced expression has been previously linked to a respiratory phenotype [20]. Moreover, our data suggest a possible modulation of miR-205 to sensitize cells to docetaxel administration.

Some papers highlighted that an increased glycolytic flux accompanied by activation of the PPP is implicated in chemoresistance of cancer cells [29, 32]. The NADPH coenzyme, produced during oxidative phase of PPP, is crucial to provide the reducing equivalents for redox reaction involved in protecting against ROS. Our data demonstrate that docetaxel-resistant PCa cells do not rely on PPP. At the same time an important physiological adaptation consists in a decrease of their ROS content. Indeed, resistant cells show low proliferation rate (Figure 1F) and do not need to fuel anabolic pathways from PPP or glycolysis, so allowing OXPHOS addiction.

In this study we analyzed the metabolic adaptation of PCa cells due to their achievement of docetaxel resistance. We observed that PC3-DR cells shift from Warburg metabolism toward mitochondrial respiration, in order to acquire a metabolic advantage. This metabolic conversion is confirmed by both down-regulation of glycolytic markers (HKII and MCT4) and increased utilization of radiolabeled carbon source (glucose, lactate and glutamine) through oxidative mitochondrial respiration (Figures 2–4). Although cancer cells are often characterized by intense glycolysis, even in the presence of oxygen (Warburg effect), mounting evidence underlines the role of OXPHOS in cancer progression and metastasis. Actually, Viale et al., demonstrated that pancreatic tumour cells, surviving oncogene ablation and responsible for tumour relapse, rely on OXPHOS for survival [39]. Sun et al., showed the existence of a shift towards OXPHOS during EMT of drug resistant lung cancer cells [40]. Acquisition of chemoresistance to temozolomide in glioma is associated with efficient mitochondrial coupling and reduced ROS production [41]. More recently, the group of Kalluri showed that the Peroxisome proliferator-activated receptor gamma coactivator 1-α (PGC-1α)-mediated mitochondrial biogenesis and respiration in cancer cells is functionally relevant for tumour cells motility and metastatic dissemination [42]. Finally, in support of our findings, Lamb et al., proposed the use of antibiotics to target mitochondria of cancer stem cells for multiple tumour types [43].

Few data are known about prostate cancer metabolism but a recent study has shown that patients bearing mitochondrial malate dehydrogenase 2 (MDH2) overexpression have shorter relapse free survival after neoadjuvant chemotherapy [44]. Moreover, inhibition of MDH2 in PCa cell lines increase the chemotherapy efficacy [44]. Our data suggest the involvement of mitochondrial respiration in the acquisition of docetaxel resistance as confirmed by ability of metformin to selectively inhibit both proliferation and invasion of resistant cells (Figure 3). Actually the inhibitory effect of metformin is not completely able to re-sensitize resistant cells to docetaxel, probably due to the existence of other compensatory mechanisms. Notably, we showed docetaxel-resistant cells as great exploiters of glutamine. Accordingly, overexpression of c-Myc was associated with sensitization to the anti-proliferative effects of metformin, consistent with c-Myc involvement in “glutamine addiction” [45]. Metformin acts directly on the mitochondria to inhibit OXPHOS and reduce mitochondrial ATP production, thus forcing tumor cells to compensate by increasing glycolysis rate [46]. Docetaxel-resistant cells - that rely heavily on OXPHOS for energy production – show a glycolytic switch and, consequently, an energy crisis associated to the loss of malignant phenotype, upon metformin treatment. For this reason, metformin could be used to exploit this metabolic weakness in resistant cells, circumventing treatment resistance and enhancing treatment efficacy. Accordingly, prostate cancer stem cells have been shown to be a good target for metformin treatment [46]. In addition, the PKM2-activator DASA-58, a well-known molecule that allows the escape from Warburg metabolism [32], is able to potentiate glucose oxidation to CO_2_ only in resistant cells, whereas sensitive cells are induced to produce lactate (Figure 2F-2G). In line, gene expression analysis retrieved from publicly available microarray analysis of PC3 cells that are resistant to docetaxel revealed an upregulation of PDHA1 mRNA expression, suggesting the increase of pyruvate oxidation in TCA cycle and the upregulation of PPARGC1A crucial for enhanced OXPHOS, mitochondrial biogenesis and oxygen consumption rate (Figure 5). Overall, it is possible that the recovery of OXPHOS subtracts intermediates from anabolic processes and from PPP, to drive ATP production. Thus, ATP may be used to enhance the activity of multi drug resistant (MDR) transporter in order to extrude the drug.

The crucial role of OXPHOS in metabolic reprogramming induced by CAF and miR-205 down-regulation [19–21] as well as in the induction of docetaxel resistance, suggested us to investigate the role of both stroma and miR-205 modulation in chemoresistance. Indeed, we demonstrated that CAFs promote the onset of PCa cells resistance to docetaxel as well as the survival of resistant tumor cells when exposed to increased concentration of the drug (Figure 6), thus playing a crucial role in protecting tumor cells against docetaxel toxicity. Accordingly, some papers show that CAFs provide an important niche for the development of drug resistant cancer cells, in part through paracrine signaling interactions with cancer cells and cancer stem cells [47, 48].

Moreover, aberrant expression of several miRNAs leads to the development of resistant prostate cells [22]. Among the others, down-regulation of miR-205 has been already linked to poor therapeutic outcome of prostate cancer patients [49]. Down-modulation of miR-205 results in upregulation of the anti-apoptotic protein Bcl2 [50] as well as down-regulation of E-cadherin, increase of Zeb 1 and Zeb2 [23, 51]. Consistently Puhr et al., demonstrated that miR-205 down-regulation is responsible for the acquisition of EMT in docetaxel resistant PCa cells [8] as well as with the acquisition of respiratory phenotype upon CAF contact [8, 20]. The close correlation between miR-205 and OXPHOS suggested us the possibility to modulate this miRNA to hinder chemoresistance. Indeed, we have shown that the ectopic expression of miR-205 reverts the OXPHOS addiction of PC3-DR, closely connected with the detoxifying ability of these cells (Figure 7). Finally, we observed that PCa cells metabolic reprogramming elicited by drug resistance is based on OXPHOS as mandatory feature of cancer malignancy. Indeed, disruption of this metabolic state by drug poisoning OXPHOS or by overexpressing miR-205 make resistant cells more sensitive to the docetaxel, implicating that drug resistance could be circumvented. The evidences highlighted in this manuscript could be relevant in a future clinical context, providing the rationale for using OXPHOS inhibitors to target metabolic reprogramming and counteract resistance to docetaxel.

Overall, our findings suggest that the combination of chemotherapy and OXPHOS inhibition may limit docetaxel-associated drug resistance and progression towards metastatic disease.

## MATERIALS AND METHODS

### Materials

Unless specified, all reagents were obtained from Sigma and all the antibodies were from Santa Cruz Biotechnology, except for anti-IL-6 (AbCam). Matrigel Matrix was purchase from BD Biosciences. [U-^14^C] lactate, [U-^14^C] glucose and [U-^14^C] glutamine were from Perkin Elmer. All kits used to perform miRNA extraction and quantitative reverse transcriptase PCR were bought from Qiagen. c-Myc siRNA (sc-29226) and Control siRNA-A (sc-37007) were from Santa Cruz. Lipofectamine 2000 and Lipofectamine RNAiMAX Reagent were from Invitrogen. Metformin was obtained from Sigma. Glutaminase Inhibitor (compound 968) was purchased by Calbiochem. Molecular Clip and Tweezer were provided by Prof. T. Schrader (University of Duisburg-Essen, Germany); DASA-58 was synthesized by Prof. C. Nativi and B. Richichi, Department of Biochemistry, University of Florence.

### Cell cultures

PC3 were purchased from the European Collection of Cell Cultures (ECACC). DU145 and DU145 docetaxel-resistant cells (12.5 nmol/L) were a kind gift of Dr. M. Puhr (Medical University of Innsbruck, Austria). PC3 cells were cultured in DMEM containing 10 % Fetal Bovine Serum (FBS). DU145 were cultured in RPMI-1640 medium supplemented with 10 % Fetal Calf Serum (FCS). CAFs were isolated from surgical explants after patients' informed consent as previously described [19], in accordance with the Ethics Committee of the Azienda Ospedaliera Universitaria Careggi, Florence. PC3 sensitive cells were converted to PC3-DR by exposing them to an initial dose of 1 nmol/L docetaxel and culturing surviving cells during 1 year with increasing doses (2.5, 5, 7.5 nmol/L) until the final concentration of 10 nmol/L, as previously described [35].

### Co-cultures separation

PC3 cells and fibroblasts were plated in a 1:2 ratio for co-culturing. Cells were then detached with Accutase (Life Technologies) and separated with MACS Column Technology (Miltenyi Biotec) by using anti-Fibroblast MicroBeads (Miltenyi Biotec) for positive selection of fibroblasts.

### Transfection

miR-205 precursor (hsa-miR-205-5p) and negative control were purchased as Pre-miR™ (Ambion). Cells were transfected using Lipofectamine-2000 following manufacturer's instructions. c-Myc siRNA and Control siRNA-A were transfected using Lipofectamine RNAiMAX Reagent according to manufacturer's instructions.

### Western blot analysis

Cells were lysed in RIPA buffer and 20-50 μg of total proteins were loaded on precast SDS-PAGE gels (BioRad). Western blot analysis was performed as previously described [18].

### Assay of intracellular reactive oxygen species (ROS)

To evaluate intracellular ROS content, cells were treated for 5 min with 5 μg/ml H_2_DCF-DA (Sigma). After PBS washing, cells were lysed in RIPA buffer and analyzed immediately by fluorimetric analysis at 510 nm. Data have been normalized to total protein content.

### Prostasphere formation

Cells were detached using Accutase (Sigma). For prostasphere formation, single cells were plated at 150 cells/cm^2^ on low attachment 100-mm plate (Corning) in DMEM/F12 (Invitrogen) supplemented with B27 and N2 (Invitrogen), 5 μg/mL insulin, 20 ng/mL basic fibroblast growth factor, and 20 ng/mL epidermal growth factor. Cells were grown under these conditions for 21 days and then prostaspheres were photographed as representative images.

### Gelatin zymography

Serum-free medium from confluent monolayer of cells was collected and 20 μL were added to the sample buffer (SDS 0.4%, 2 % glycerol, 10 mmol/L Tris-HCl, pH 6.8, 0.001 % bromphenol blue). The sample was run on a 10 % SDS gel containing 0.1 % gelatin. After electrophoresis, the gel was washed twice with 2.5 % Triton X-100 and once with reaction buffer (50 mmol/L Tris-HCl (pH 7.5), 200 mmol/L NaCl, 5 mmol/L CaCl_2_). The gel was incubated overnight at 37°C with freshly added reaction buffer and stained with Laemli Comassie blue solution. Areas of gelatinase activity appear as clear bands against a dark background.

### *In vitro* boyden invasion assay

Invasion assay was performed with 8 × 10^4^ cells on 8-μm-pore Transwells (Corning) coated with 50 μg/cm^2^ of reconstituted Matrigel for 16 h, as described in [52]. Chemotaxis was evaluated by counting the cells migrated to the lower surface of the filters (six randomly chosen fields).

### Cell treatments and cell growth assay

After plating cells, medium was changed with fresh medium containing either glucose, sodium pyruvate or glutamine (25, 1 or 2 mmol/L) or both in the absence or presence of metformin (5 mmol/L), compound 968 (10 μmol/L), N-acetylcysteine (20 mmol/L), antimycin (1 μmol/L), oligomycin (500 nmol/L) or rotenone (5μmol/L) for another 12, 48, or 72 h, depending on the experiment. Cell growth was determined by crystal violet staining. At indicated times, cells were washed twice with PBS and crystal violet solution (0.5 % in 20 % methanol) was added and incubated for 15 min. The remaining crystals were dissolved with 0.1 M sodium citrate (pH 4.2). Viability was determined by absorbance at 595 nm using a spectrophotometric reader.

### Real time RT-PCR

Total RNA from PC3 cells was extracted using RNeasy (Qiagen) according to the manufacturer's instructions. Strands of cDNA were synthesized using QuantiTect Reverse Transcription Kit (Qiagen) using 1 μg of total RNA. mRNA expression was performed using QuantiFast SYBR Green (Qiagen). The primers for *ZEB1* were: 5′-AGCAGTGAAAGAGAAGGGAATGC-3′(forward), 5′ GGTCCTCTTCAGGTGCCTCAG-3′ (reverse); for *ZEB2*: 5′-GGCATATGGTGACGCACAA-3′ (forward), 5′-TTGAACTTGCGGTTACCTGC-3′ (reverse); for *GLUT1* 5′-CGGGCCAAGAGTGTGCTAAA-3′ (forward), 5′-TGACGATACCGGAGCCAATG-3′ (reverse). Data were normalized on β-2 microglobulin. Results are shown as the mean of three different experiments ±SD. For quantification of miR-205 expression levels, total RNA, including small RNAs, was purified using miRNeasy kit. The reverse transcription reaction of 1 μg of total RNA was carried on using miScript II RT kit and the quantification of miR-205 expression level was assessed by Real Time PCR using miScript SYBR Green PCR kit and miScript Primer Assay-HsmiR-205. SNORD61 was used as normalizer (miScript Primer Assay-HsSNORD61, Qiagen). All the amplifications were run on 7500 Fast Real-Time PCR System. Data were reported as relative quantity with respect to the calibrator sample using the 2^−ΔΔCt^ method.

### Lactate assay

Lactate levels were determined using a lactate assay kit (BioVision) according to the manufacturer's instructions. All data were normalized on cell protein content.

### Radioactive metabolic assays

A) Radioactive glucose, lactate or glutamine uptake was evaluated by treating cells with a buffered solution (140 mmol/L NaCl, 20 mmol/L Hepes/Na, 2.5 mmol/L MgSO4, 1 mmol/L CaCl_2_, and 5 mmol/L KCl, pH 7.4) containing 0.5 μCi/mL [U-^14^C] glucose, [U-^14^C] lactate [U-^14^C] glutamine for 15 min at 37°C. Cells were subsequently washed with cold PBS and lysed with 0.1 mol/L NaOH. Incorporated radioactive was assayed by liquid scintillation counting and normalized on protein content. B) For the detection of CO_2_ released from radioactive substrates, cells were incubated with 0.2 μCi/mL or 0.5μ Ci/mL [U-^14^C] lactate or [U-^14^C]-glucose and glutamine for 15 min into 35 mm-dish. Each dish had a taped piece of Whatman paper facing the inside of the dish wetted with 100 μL of phenyl-ethylamine-methanol (1:1) to trap the CO_2_. Then 200 μL of 4M H_2_SO_4_ was added to cells. Finally, Whatman paper was removed and transferred to scintillation vials for counting. C) PPP activity was evaluated by using [1-^14^C]-glucose and [6-^14^C]-glucose. ^14^CO_2_ developed from [1-^14^C]-glucose oxidation originates by the PPP or by the TCA cycle, whereas ^14^CO_2_ released from [6-^14^C]-glucose originates only by TCA cycle. 2 μCi [1-^14^C]-glucose or 2 μCi [6-^14^C]-glucose were added for 1 h to cells, in two different plates of same sample. Then, CO_2_ released was measured as above. The extent of PPP metabolic flux was obtained by subtracting the amount of CO_2_ developed from [6-^14^C]-glucose from the CO_2_ released from [1-^14^C]-glucose.

### G6PD activity assay

Cell lysis was performed at 4°C in 50 mM Tris/Hcl pH 7.4, containing protease inhibitors. After 30 min of incubation on ice, lysates were sonicated and centrifuged at 12,000 *g* in a microcentrifuge at 4°C for 30 min. Supernatants were quantified with respect to protein content by Bradford assay (Biorad). G6PD activity was assayed following NADPH absorbance at 340 nm over 20 min (ε = 6.22 mM^−1^ cm^−1^).

### Evaluation of NADPH level

NADPH levels were determined using a NADP/NADPH Quantitation Colorimetric Kit (BioVision) according to the manufacturer's instructions. All data were normalized on protein content.

### Evaluation of apoptotic cell death

Cells were detached, washed with PBS and resuspended in 100 μl of buffer solution (Thermo Fischer Scientific) containing 1μl of Annexin V and 1μl of Propidium Iodide. After 15 min incubation at room temperature, cell staining was evaluated by flow cytometry, gating for positivity to Annexin V and/or Propidium Iodide.

### Gene expression analysis of publicly available datasets

GDS3973 data [35] were retrieved from GEO profiles. The log^2^ transformed counts were obtained mapping SLC16A4 (205234_at), SLC16A1 (202235_at), HK2 (222305_at), PDHA1 (1555864_s_at), TIGAR (219099_at), MIR-205HG (226755_at), DLD (230426_at), MYC (244089_at), PPARGC1A (1569141_a_at), SNAI2 (213139_at), ZEB1 (212764_at), VIM (201426_s_at), TWIST2 (229404_at), CDH1 (201131_s_at). Statistical analysis was performed using Prism software (GraphPad version 6.0 Software, La Jolla, CA, USA). Data were compared using Student's t-test.

### Statistical analysis

Data are presented as means ± SD from at least three independent experiments. Statistical analysis of the data was performed by Student's t test or 2-way ANOVA (Bonferroni corrected). P values of ≤0.05 were considered statistically significant.

## SUPPLEMENTARY FIGURES


