# Supplementary material for: Metabolic shift toward oxidative phosphorylation in docetaxel resistant prostate cancer cells
Source: Oncotarget. 2016 Aug 16;7(38):61890–904. doi: 10.18632/oncotarget.11301 (PMC5308698; doi:10.18632/oncotarget.11301)
Supplement: Supplementary file 1 [file oncotarget-07-61890-s001.pdf]

Metabolic shift toward oxidative phosphorylation in docetaxel resistant prostate cancer cells

SUPPLEMENTARY FIGURES

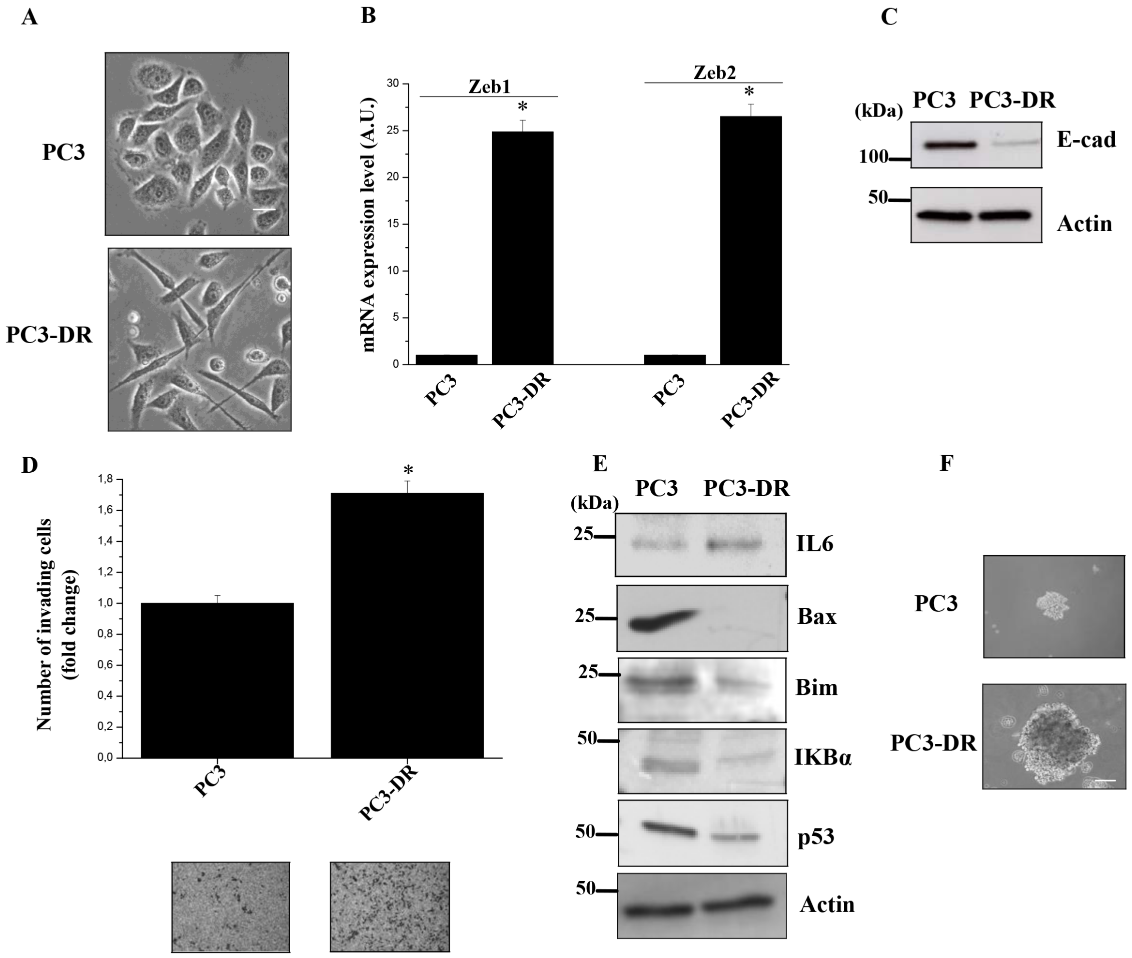

Supplementary Figure S1: Docetaxel resistant cells undergo EMT, increase invasion and acquire stem cell feature. A. Pictures of PC3 and PC3-DR cells were taken. Bar, 30 µm. **B.** Total RNA was extracted and Zeb1 and Zeb2 mRNA expression levels were analyzed by qRT-PCR. Results are representative of three independent experiments. *p<0.005 PC3-DR *vs* PC3. **C.** Expression levels of E-cadherin in PC3 and PC3-DR cells were evaluated by westen blotting. Actin immunoblot was used to ensure equal protein loading. **D.** Invasion assay using Matrigel-coated Boyden chamber was performed using PC3 and PC3-DR cells. Representative pictures are shown and quantification of six randomly chosen fields (n=3) is reported. *p<0.01 PC3-DR *vs* PC3. **E.** Expression levels of secreted IL6, Bax, Bim, IkBα, p53 in PC3 and PC3-DR cells. Actin immunoblot was used to ensure equal protein loading. **F.** Representative images of clones obtained from PC3 and PC3-DR cells after 21 days of cell culture as describe in Material and Methods. Bar, 100 µm.

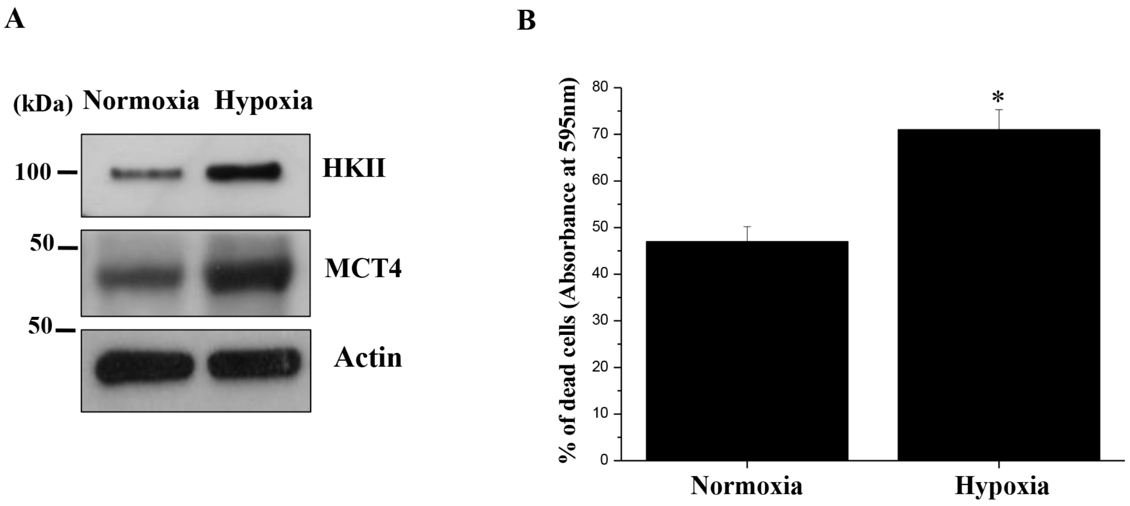

Supplementary Figure S2: Hypoxic conditions confer to PC3-DR cells an increase in chemosensitivity. A. Expression levels of HKII and MCT4 in PC3-DR cells kept in normoxic or hypoxic (1% O_2) conditions for 24 h. Actin immunoblot was used to ensure equal protein loading. **B.** PC3-DR cells were kept in normoxia or hypoxia for 24 h in the presence or not of 20 nM docetaxel. Cells were then quantified by crystal violet assay. Bar graph represents the percentage of dead cells following docetaxel treatment with respect to untreated cells. Results are representative of three experiments. *p<0.005 hypoxia *vs* normoxia.

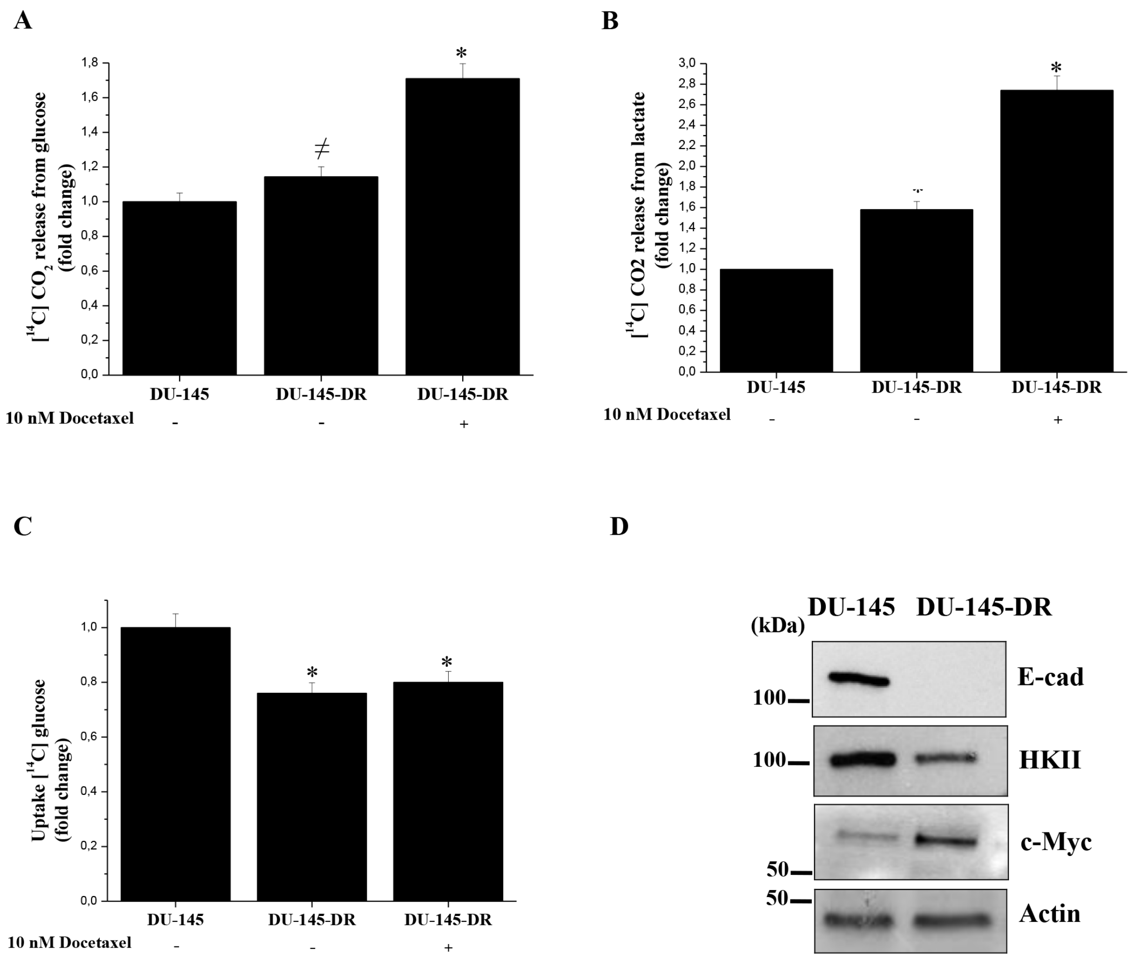

Supplementary Figure S3: DU-145-DR cells increase OXPHOS respect to sensitive cells. A-B. Respiration of [^{14}C]-glucose and [^{14}C]-lactate of DU-145 and DU-145-DR cells treated with or without 10 nM docetaxel for 48 h in serum free medium. Respiration was evaluated by monitoring [^{14}C]-CO_2 release and normalized on protein content. Results are shown as fold change relative to DU-145-DR. ≠p<0.05 *vs* DU-145; *p <0.005 *vs* DU-145. **C.** Cells were treated as in A-B) and the evaluation of [^{14}C]-glucose uptake was performed and normalized on protein content. *p<0.005 DU-145-DR cells *vs* DU-145. **D.** Expression levels of E-cadherin, HKII and c-Myc in DU-145 and DU-145-DR cells. Actin immunoblot was used to ensure equal protein loading.

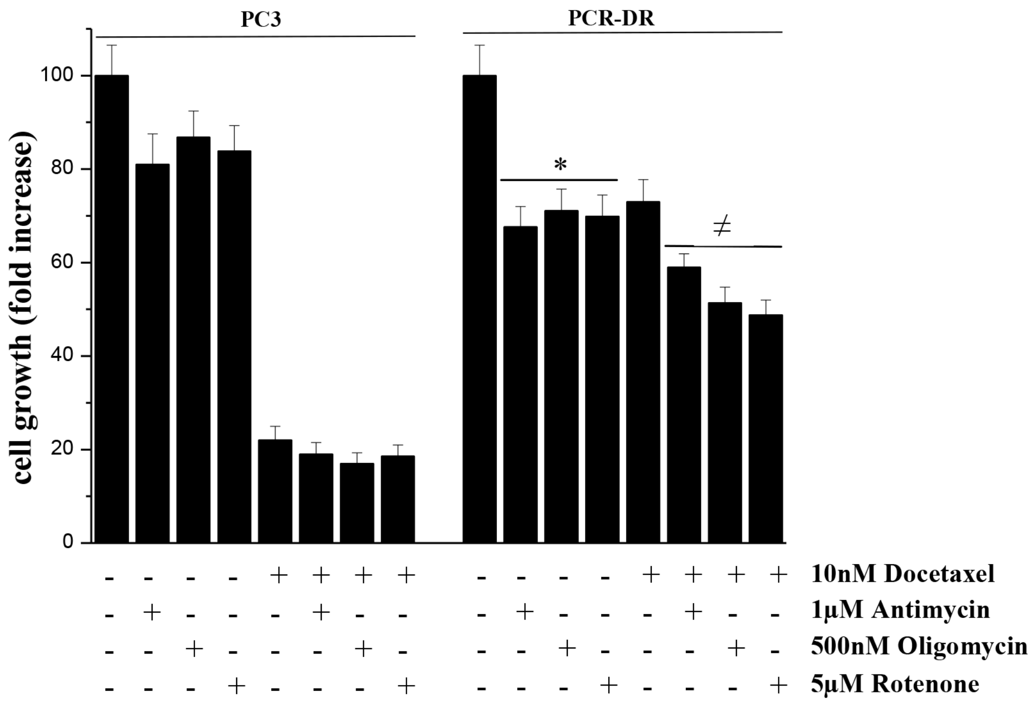

Supplementary Figure S4: OXPHOS inhibitors decrease growth of PC3-DR cells. Analysis of PC3 and PC3-DR cells growth in presence of 10 nM docetaxel and/or 1μM antimycin, 500nM oligomycin or 5μM rotenone. OXPHOS inhibitors were added 30 min before docetaxel. After 48 h cell growth was assayed by crystal violet staining. Absorbance at T0 of untreated PC3 and untreated PC3-DR cells was used as control. The results are representative of three experiments. *p<0.005 OXPHOS inhibitors *vs* control. ≠p<0.005 OXPHOS inhibitors/docetaxel treated *vs* docetaxel treated.

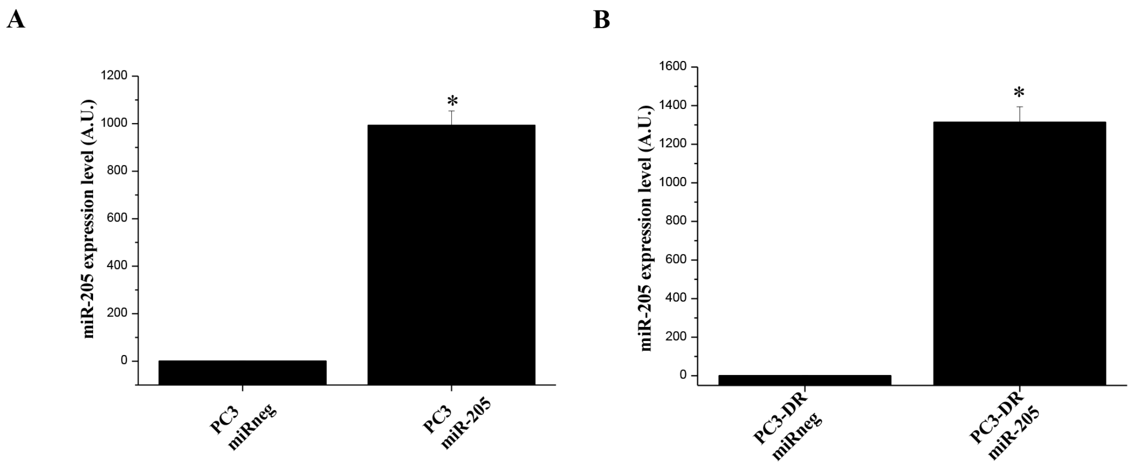

Supplementary Figure S5: Ectopic expression of miR-205 in prostate cancer cells. PC3 cells **A.** or PC3-DR cells **B.** were transfected with miRneg or miR-205. After 48 h from transfection, total RNA was extracted from cells and miR-205 expression levels were analyzed by qRT-PCR. Results are representative of three independent experiments. *p<0.005 miR-205 trasfected *vs* miRneg trasfected.

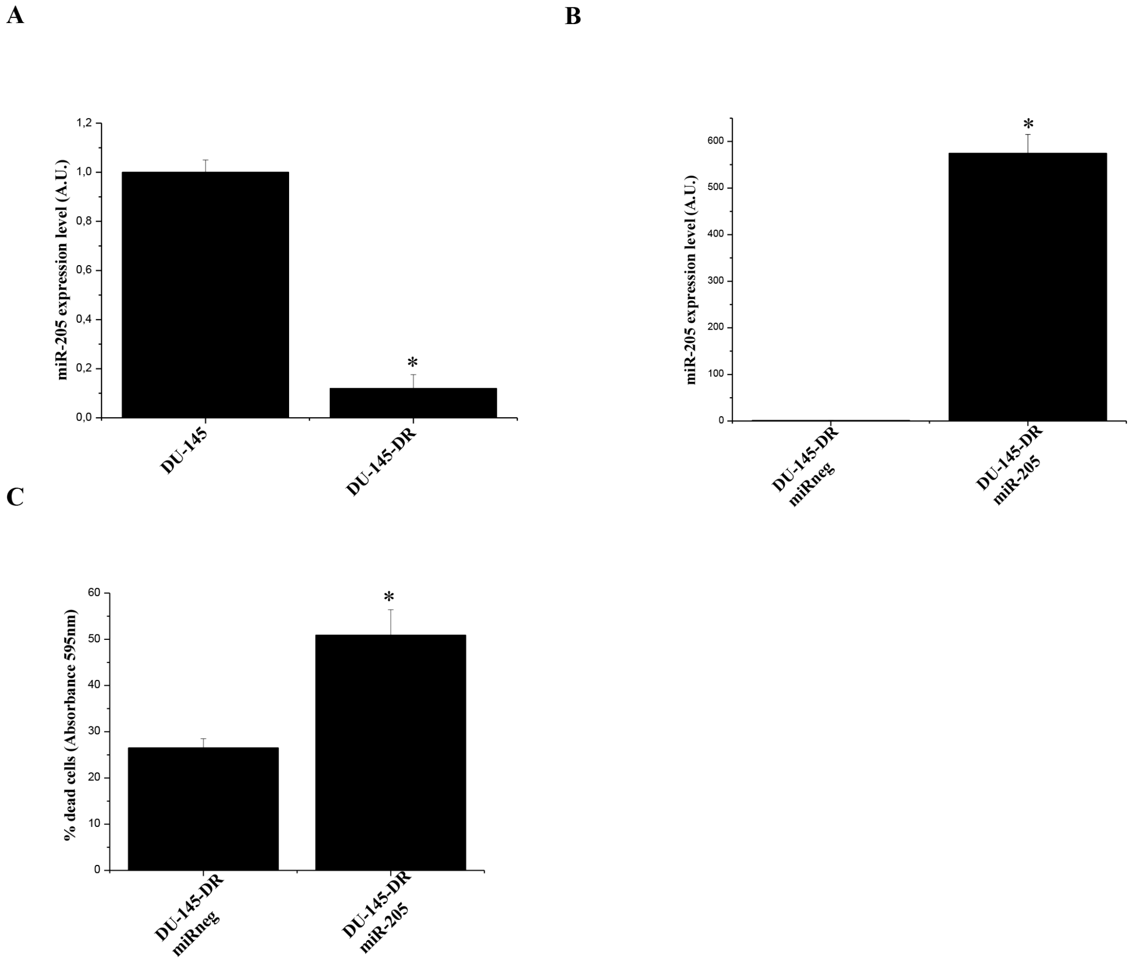

Supplementary Figure S6: Ectopic expression of miR-205 sensitizes DU-145-DR cells to docetaxel. A. Total RNA was extracted from DU-145 and DU-145-DR cells and miR-205 expression levels were analyzed by qRT-PCR. Results are representative of three independent experiments. *$p<0.005$ DU-145-DR *vs* DU-145. **B.** DU-145-DR cells were transfected with miR-205 or miRneg for 48 h and then miR-205 expression levels were analyzed by qRT-PCR. Results are representative of three independent. *$p<0.005$ miR-205 transfected cells *vs* miRneg transfected cells. **C.** DU-145-DR cells were transfected with miR-205 or miRneg for 24 h and then treated with or without 40 nM docetaxel for 48 h. Cells were then quantified by crystal violet assay. Bar graph represents the percentage of dead cells following docetaxel treatment relative to untreated cells. Results are representative of four experiments. *$p<0.005$ miR-205 transfected DU-145-DR *vs* miRneg transfected DU-145-DR.
